# Age and Racial/Ethnic Differences in Dietary Sources of Protein, NHANES, 2011–2016

**DOI:** 10.3389/fnut.2020.00076

**Published:** 2020-06-26

**Authors:** Jeannette M. Beasley, Melanie J. Firestone, Collin J. Popp, Rienna Russo, Stella S. Yi

**Affiliations:** ^1^Department of Medicine, NYU Langone Health, New York, NY, United States; ^2^School of Public Health, University of Minnesota, Minneapolis, MN, United States; ^3^Department of Population Health, NYU Langone Health, New York, NY, United States

**Keywords:** nutrition, epidemiology, aging, Asian American, African American, Hispanic American

## Abstract

**Background:** Dietary protein serves a pivotal role in providing the body with essential amino acids, which are required for the maintenance of body proteins, and the assimilation of structural and functional components required for basic survival. Understanding how dietary protein sources potentially vary for different population subgroups will allow for future nutrition interventions to be more targeted for specific needs.

**Objective:** The purpose of this analysis was to identify the top ten food category sources of dietary protein by age and race and ethnicity in a nationally representative sample.

**Methods:** Cross-sectional data on adults (18+ years) from the National Health and Nutrition Examination Survey (NHANES) 2011–2016 with one 24-h dietary recall were analyzed (*n* = 15,697). Population proportions were calculated based on protein intake (g/day) for What We Eat In America food categories.

**Results:** The analytic sample (*n* = 15,697) was 15.0% Hispanic (95% CI [12.1, 17.9], 65.0% non-Hispanic White (95% CI [60.8, 69.3]), 11.5% non-Hispanic Black (95% CI [9.1, 13.9]), 5.4% non-Hispanic Asian (95% CI [4.3, 6.6]), and 3.1% other (95% CI [2.5, 3.6]). In all racial and ethnic groups, as well as age categories, chicken (whole pieces) was the top-ranked source of dietary protein. In addition to chicken (whole pieces), beef (excludes ground), eggs and omelets, and meat mixed dishes food categories ranked in the top ten sources of protein for every race/ethnicity. Only two solely plant-based proteins appeared in the top ten sources: beans, peas and legumes for Hispanics, and nuts and seeds for Other. For all age categories, beef (excludes ground) was among the top five sources and egg/omelets appear in the top ten sources.

**Conclusion:** The top ten sources of protein accounted for over 40% of dietary protein irrespective of race/ethnicity or age category, having major implications for the sustainability of our nation's food supply. Public health strategies that encourage diversity in protein sources in food preparation and incorporate legumes and nuts along with poultry have the potential to shift the overall population protein intake distribution toward improving overall diet quality.

## Introduction

Dietary protein serves a pivotal role in providing the body with essential amino acids, which are required for the maintenance of body proteins, and the assimilation of structural and functional components required for basic survival (1). Protein is unique in comparison to carbohydrates and fats in that it is a nitrogen-containing compound. Under normal conditions, protein is not stored for the purposes of energy production, as are carbohydrates or fatty acids. Furthermore, dietary protein is uniquely metabolized, resulting in a higher postprandial thermic effect of food compared to carbohydrates and fats. For those purposes, dietary protein is also more satiating, leading to postprandial reductions in hunger (2, 3). Protein-rich diets have been found to maintain muscle mass, increase weight loss, and improve metabolic function across the lifespan (4).

Dietary protein intake is important across the lifespan, in particular among older adults. The Recommended Dietary Allowance (RDA) for protein is 0.8 g/kg/day for all adults, and age- and gender- specific National Health and Nutrition Examination Survey (NHANES) analyses reported while 3% of men ages 19–30 consumed less than the RDA, 19% of women ages 71 and older consumed less than the RDA (5). Age-associated declines in muscle function, muscle wasting, frailty, and reduced quality of life have been well-documented (6–8). Optimal intake of dietary protein during aging may help alleviate the decrease in muscle mass and maintain the functional integrity of body proteins (9). Despite this, the proportion of older adults meeting the RDA for protein is mixed (5, 10, 11).

The degree to which protein needs are not being met not only varies by age but also by race/ethnicity. Prior assessment of protein intake suggests that Asian American populations have higher intakes of protein as a percentage of calories compared to non-Hispanic White (NHW) and non-Hispanic Black (NHB) populations (5, 12). The higher intakes of protein were also observed in older, Asian American adults, with 17% protein as a percent of total calories in both the >51 years and ≥71 years categories (5). In comparison, older NHW adults consume roughly 15% of their total calories from protein. While we know trends since 1999 are toward an older (increase from 18 to 21.1% of adults aged ≥65 years) and more diverse (proportion who were non-Hispanic white declined from 69.8 to 64.4%) population (13), we hypothesize, that dietary protein sources vary by age and race/ethnicity. Understanding dietary protein sources can help inform intervention efforts addressing protein intake to accommodate racial/ethnic diversity in the United States. Therefore, the purpose of this analysis was to identify the top ten food category sources of dietary protein by age and race and ethnicity in a nationally representative sample.

## Materials and Methods

### Study Design

The National Health and Nutrition Examination Survey (NHANES) is a program of studies designed to assess the health and nutritional status of non-institutionalized, civilian adults in the United States (14). Those who participate in the NHANES provide demographic and general health information followed by a visit to a Mobile Examination Center for anthropometric measurements and 24-h dietary recalls, which are conducted by a trained interviewer using the USDA-multiple pass method. The Research Ethics Review Board at the National Center for Health Statistics approved all survey protocols, and all participants and their proxies provided written informed consent.

The USDA Food and Nutrition Database for Dietary Studies (FNDDS) provides nutrient values for foods and beverages reported in each 24-h dietary recall based on the USDA National Nutrient Database for Standard Reference (15). FNDDS food categories were linked to one What We Eat in America (WWEIA) category, a classification scheme that includes ~150 food categories and does not disaggregate into ingredients (i.e., pizza vs. cheese, tomatoes, etc.), to analyze protein intake in the previous 24-h from food and beverages (16). The categorization is designed to group similar foods and beverages together based on usage and nutrients. Meat mixed dishes, for example, typically include meat served with a sauce (i.e., meat with gravy) or with vegetables (i.e., beef stew). The purpose in using these categories is to represent how these foods are typically consumed.

Data from a single 24-h recall for three waves, 2011–2016, were pooled as these were the years during which non-Hispanic Asian Americans were oversampled (17). The resulting dataset had a sample size of 29,902. Participants were excluded if they were <18 years of age (*n* = 11,933), were missing the dietary recall (*n* = 2,271), or reported consuming exclusively water (*n* = 1). The final analytic sample included 15,697 individuals.

### Measures

Descriptive demographic variables included sex, age, race/ethnicity, education, and nativity. Race/ethnicity was divided into five categories: Hispanic, non-Hispanic White (NHW), non-Hispanic Black (NHB), non-Hispanic Asian (NHA), and Other (includes mixed race). Education was restricted to adults ≥25 years. Age was divided into four categories: 18–24, 25–44, 45–64, and ≥65 years. The race/ethnicity categories and age categories were further combined so that within each race/ethnicity category there were four age-specific categories. For example, NHW was divided into (1) NHW, 18–24; (2) NHW, 25–44; (3) NHW, 45–64; and (4) NHW, ≥65 years.

Means and standard errors for protein intake (g/d), energy intake (kcal/d), and protein density (%kcal/d) were calculated by age and race/ethnicity. To determine the top ten dietary sources of protein, population proportions (%) were calculated for each food category by summing the amount of protein consumed within each category for all persons within each subcategory (age, race/ethnicity) and dividing that by the sum of all protein consumed for all foods for all persons within each subcategory (age, race/ethnicity) multiplied by 100 (18).

### Statistical Analyses

Food categories were ranked based on population proportion and the top ten are reported. Mean differences comparing Asian Americans to other races/ethnicities and adults ages ≥65 to other age groups were calculated using *t*-tests. Tests were considered statistically significant if *p* < 0.05. Correction for multiple comparisons was not conducted given the small number of planned comparisons. Calculations were estimated using survey procedures in SAS v.9.4 (SAS Institute Inc, Cary, NC) to adjust for the complex survey design. Sample weights accounted for the probability of selection, non-response, and day of the week of dietary recall.

## Results

The analytic sample (*n* = 15,697) was 15.0% Hispanic (95% CI: [12.1, 17.9]), 65.0% NHW (95% CI: [60.8, 69.3]), 11.5% NHB (95% CI: [9.1, 13.9]), 5.4% NHA (95% CI: [4.3, 6.6]), and 3.1% Other (95% CI: [2.5, 3.6]) (Table 1). Older adults (≥65 y) accounted for 18.5% of the sample. Approximately half of the sample was female (51.3%), one-third had a college degree or greater, and over four-fifths of the sample was US-born (82.8%).

**Table 1:** Demographic characteristics, NHANES 2011–2016 (*n* = 15,697).

	**Unweighted (*n*)**	**Unweighted (%)**	**Weighted (*n*)**	**Weighted (%)**	**95% CI**
Total			237,484,333		
Gender					
Male	7,635	48.6	115,541,675	48.7	47.7, 49.6
Female	8,062	51.4	121,942,658	51.3	50.4, 52.3
Age Group (yr)					
18–24	2,160	13.8	30,748,424	12.9	11.2, 14.7
25–44	5,098	32.5	79,754,209	33.6	31.6, 35.6
45–64	5,091	32.4	83,163,005	35.0	33.5, 36.5
≥65	3,348	21.3	43,818,694	18.5	17.3, 19.6
Race/Ethnicity					
Hispanic	3,869	24.6	35,560,978	15.0	12.1, 17.9
NH White	5,982	38.1	154,384,639	65.0	60.8, 69.3
NH Black	3,568	22.7	27,310,549	11.5	9.1, 13.9
NH Asian	1750	11.1	12,938,968	5.4	4.3, 6.6
Other	528	3.4	7,289,200	3.1	2.5, 3.6
Education^a^					
Less than High School	3,083	22.8	31,362,695	15.2	13.2, 17.1
High School	2,922	21.6	42,854,471	20.7	19.2, 22.3
Some College	3,908	28.9	64,239,306	31.1	29.6, 32.6
College Graduate or more	3,616	26.7	68,223,928	33.0	29.8, 36.2
Nativity					
US born	11,138	71.0	196,614,166	82.8	80.7, 85.0
Foreign born	4,551	29.0	40,773,632	17.2	15.0, 19.3

*NH, Non-Hispanic*.

a *Education restricted to ≥25 yr*.

### Protein by Race/Ethnicity

**Figure 1:**
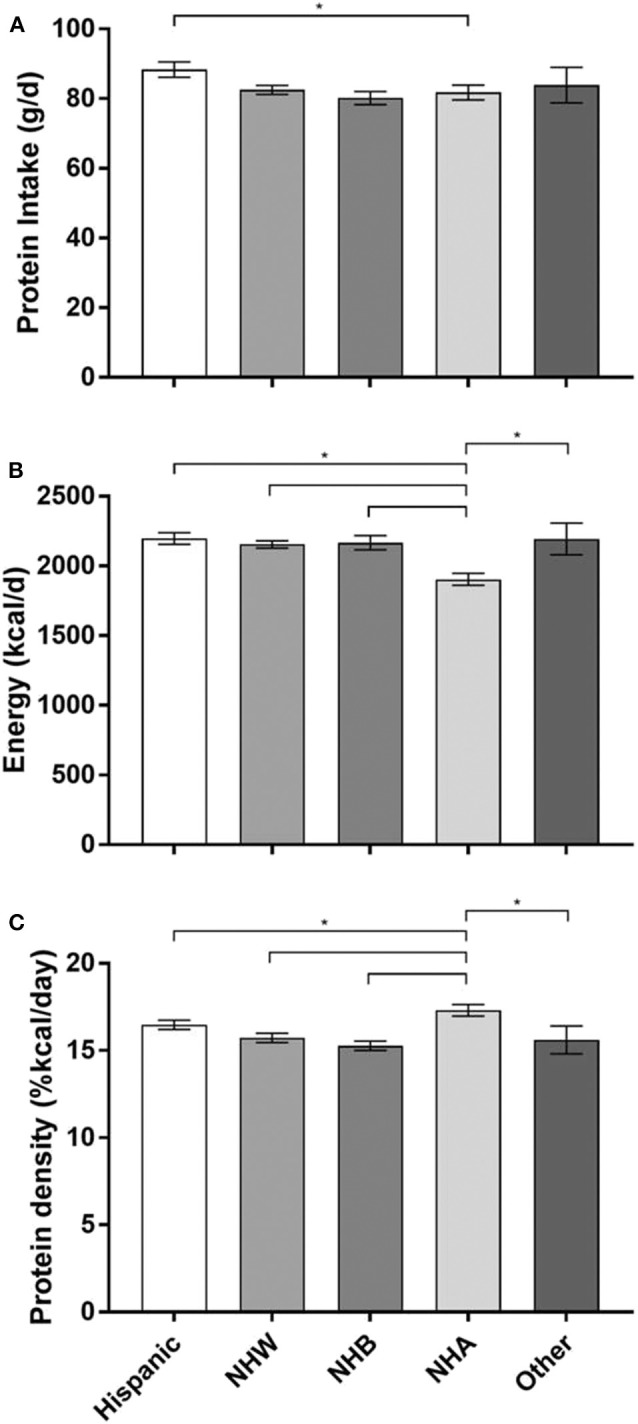
Protein intake, energy intake, and protein density by race/ethnicity. **(A)** Protein intake in grams per day (g/d). **(B)** Energy intake in kilocalories per day (kcal/d). **(C)** Protein density (%kcals/day). The three panels **(A–C)** each share the same x-axis description that are only labled on panel C.Data are reported at mean and upper and lower limits. *p < 0.05 Non-Hispanic Asian are the reference group; NHW, Non-Hispanic White; NHB, Non-Hispanic Black; NHA, Non-Hispanic Asian.

Hispanics consumed the greatest amount of absolute protein, with a mean intake of 88 grams per day (95% CI: [86, 91], *p* = 0.0003) (Figure 1). NHA consumed the fewest calories (1904, 95% CI: [1861, 1947]) compared to other race/ethnic groups (*p*-value for all < 0.0001). Therefore, protein density (%kcal/day) was significantly higher among NHA (17.3, 95% CI: [17.0, 17.6]) compared to NHB (15.3, 95% CI: [15.0, 15.5]), Other (15.6, 95% CI: [14.8, 16.4]), NHW (15.7, 95% CI: [15.5, 16.0]), and Hispanics (16.5, 95% CI: [16.2, 16.7]) (*p*-value for all < 0.001).

Adults from all race/ethnicity groups consumed at least 40% of their total protein intake from the top ten food categories. NHA consumed the most, with almost half (48.6%) from the top ten food categories, compared to 41.0% for NHW, 41.5% for Other, 45.7% for Hispanics, and 45.9% for NHB (Table 2). Across all race/ethnicity groups, chicken (whole pieces) was the top-ranked source for dietary protein (Table 2). As the top source, chicken accounted for ≤ 10% of total protein in all race/ethnicity groups except for the NHB population, where chicken accounted for 14% of total protein. Beef (excludes ground), eggs and omelets, and meat mixed dishes food categories were also ranked in the top ten sources of protein for every race/ethnicity. Pizza ranked as a top ten source of protein among all race/ethnic groups except for NHA. Fish was in the top 10 sources of protein for Hispanic, NHW, NHB, NHA, and Other. More specifically, fish was the second and third ranked source of protein for NHB and NHA, respectively. Each race/ethnicity group had at least one food category that uniquely ranked as a top ten source (Hispanic: other

**Table 2:** Protein sources by race/ethnicity.

**Rank**	**Food category**	**% Total protein**	**Cumulative %**
**Race/ethnicity**	**Hispanic**		
1	Chicken, whole pieces	9.7	9.7
2	Burritos and tacos	7.0	16.7
3	Beef, excludes ground	5.5	22.2
4	Eggs and omelets	4.3	26.5
5	Other Mexican mixed dishes	3.8	30.3
6	Pizza	3.5	33.8
7	Soups	3.2	37.0
8	Meat mixed dishes	3.1	40.1
9	Beans, peas, legumes	2.8	42.9
10	Fish	2.8	45.7
**Race/ethnicity**	**Non-Hispanic White**		
1	Chicken, whole pieces	7.1	7.1
2	Pizza	4.6	11.6
3	Cold cuts and cured meats	4.2	15.8
4	Beef, excludes ground	4.1	19.9
5	Cheese	4.1	24.0
6	Meat mixed dishes	3.8	27.9
7	Yeast breads	3.6	31.5
8	Eggs and omelets	3.5	35.0
9	Burritos and tacos	3.2	38.2
10	Fish	2.8	41.0
**Race/ethnicity**	**Non-Hispanic Black**		
1	Chicken, whole pieces	14.0	14.0
2	Fish	4.9	18.9
3	Pizza	4.1	23.0
4	Beef, excludes ground	4.0	27.0
5	Eggs and omelets	3.5	30.6
6	Meat mixed dishes	3.3	33.9
7	Burgers (single code)	3.3	37.2
8	Yeast breads	3.2	40.4
9	Pork	2.9	43.2
10	Pasta mixed dishes, excludes macaroni and cheese	2.6	45.9
**Race/ethnicity**	**Non-Hispanic Asian**		
1	Chicken, whole pieces	9.4	9.4
2	Soups	6.7	16.1
3	Fish	6.2	22.3
4	Rice	4.4	26.7
5	Stir-fry and soy-based sauce mixtures	4.4	31.1
6	Yeast breads	4.1	35.2
7	Beef, excludes ground	3.5	38.7
8	Eggs and omelets	3.3	42.0
9	Meat mixed dishes	3.2	45.2
10	Pork	3.2	48.4
**Race/ethnicity**	**Other**		
1	Chicken, whole pieces	9.3	9.3
2	Beef, excludes ground	5.6	15.0
3	Pizza	4.6	19.5
4	Fish	3.5	23.0
5	Eggs and omelets	3.4	26.5
6	Yeast breads	3.3	29.7
7	Meat mixed dishes	3.2	32.9
8	Cold cuts and cured meats	3.1	36.0
9	Nuts and seeds	2.8	38.8
10	Pasta mixed dishes, excludes macaroni and cheese	2.8	41.5

Mexican mixed dishes; NHW: cheese; NHB: burger [single code]; NHA: rice, stir-fry and soy-based dishes, and Other: nuts and seeds). Only two solely plant-based proteins appeared in the top ten sources: beans, peas and legumes for Hispanics, and nuts and seeds for Other.

### Protein by Age

**Figure 2:**
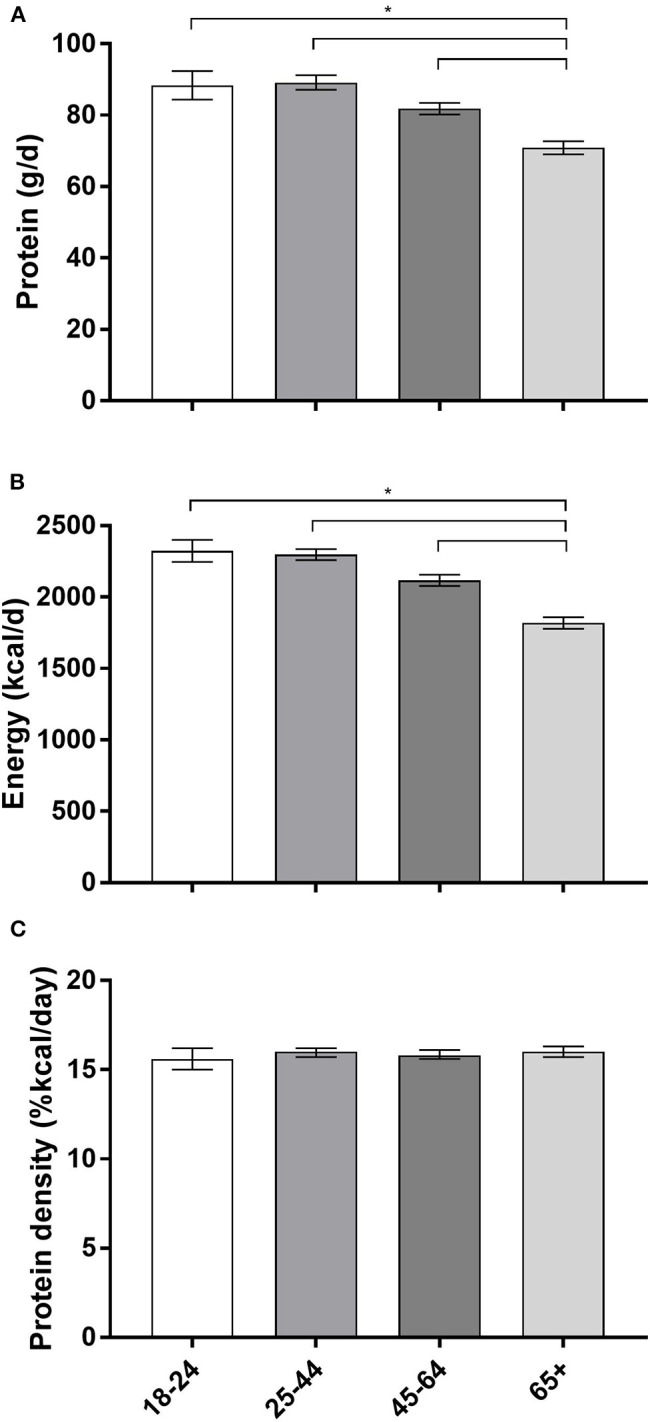
Protein intake, energy intake and protein density by age category (years). **(A)** Protein intake in grams per day (g/d). **(B)** Energy intake in kilocalories per day (kcal/d). **(C)** Protein density (%kcals/day). The three panels **(A–C)** each share the same x-axis description that are only labled on **(C)**. Data are reported at mean and upper and lower limits; data reported in years; *p < 0.05; ≥65 is the reference group.

Older adults consumed the least absolute protein (71 grams per day, 95% CI: [69, 73]) and energy (1819 calories, 95% CI: [1779, 1860] compared to other age groups (*p*-value for all < 0.0001) (Figure 2). However, protein density (%kcal/day) did not vary significantly by age group (*p*-value for all>0.05).

Chicken (whole pieces) was the top source of protein irrespective of age (Table 3). Beef (excludes ground) was among the top five sources of protein, and cheese, eggs/omelets, as well as cold cuts and cured meats, appeared in the top ten sources for all age categories. Fish was a top ten source of protein among all age categories except for 18–24 year olds. The youngest (18–24 years) and oldest (≥65 years) age categories each had two top protein sources that were exclusive to their age category: burgers and pasta mixed dishes (excluding macaroni and cheese) for 18–24 year olds, and nuts/seeds and soups for ≥65 year olds.

**Table 3:** Protein sources by age category.

**Rank**	**Food category**	**% Total protein**	**Cumulative %**
**Age category**	**18–24 years**		
1	Chicken, whole pieces	9.4	9.4
2	Pizza	7.0	16.4
3	Burritos and tacos	4.3	20.7
4	Cheese	4.0	24.6
5	Beef, excludes ground	3.8	28.5
6	Eggs and omelets	3.4	31.9
7	Cold cuts and cured meats	3.3	35.2
8	Burgers (single code)	3.0	38.2
9	Meat mixed dishes	2.9	41.2
10	Pasta mixed dishes, excludes macaroni and cheese	2.9	44.1
	**25–44 years**		
1	Chicken, whole pieces	9.3	9.3
2	Pizza	5.0	14.3
3	Beef, excludes ground	4.6	18.9
4	Burritos and tacos	4.2	23.1
5	Cheese	3.6	26.7
6	Eggs and omelets	3.6	30.3
7	Cold cuts and cured meats	3.5	33.8
8	Meat mixed dishes	3.1	36.9
9	Yeast breads	2.8	39.7
10	Fish	2.8	42.5
	**45–64 years**		
1	Chicken, whole pieces	7.8	7.8
2	Beef, excludes ground	4.5	12.3
3	Meat mixed dishes	4.0	16.3
4	Fish	3.8	20.1
5	Yeast breads	3.8	23.9
6	Eggs and omelets	3.6	27.6
7	Pizza	3.5	31.1
8	Cold cuts and cured meats	3.4	34.5
9	Cheese	3.4	38.0
10	Burritos and tacos	2.9	40.9
	≥**65 years**		
1	Chicken, whole pieces	7.0	7.0
2	Yeast breads	5.0	11.9
3	Meat mixed dishes	4.5	16.5
4	Eggs and omelets	3.9	20.4
5	Beef, excludes ground	3.9	24.2
6	Fish	3.6	27.8
7	Cold cuts and cured meats	3.6	31.4
8	Nuts and seeds	3.1	34.5
9	Cheese	2.9	37.5
10	Soups	2.8	40.3

### Protein by Race/Ethnicity and Age

Chicken (whole pieces) was the only protein source to appear as a top protein source in all categories, irrespective of race/ethnicity and age (Table 4). While pizza appeared as a top protein source for all race/ethnic groups under age 45, it was not a top protein source for any race/ethnic group among those aged ≥65 years. In contrast, fish was a top protein source for all/race/ethnic groups over age 44 years, but it was only a top protein source among NHB in the 18–24 year old age category.

**Table 4:** Protein sources by race/ethnicity and age category.

**Rank**	**Food category**	**% Total protein**	**Cumulative %**
	**Hispanic: 18–24 years**		
1	Chicken, whole pieces	10.5	10.5
2	Burritos and tacos	7.7	18.2
3	Pizza	6.2	24.4
4	Beef, excludes ground	5.0	29.3
5	Burgers (single code)	3.8	33.2
6	Meat mixed dishes	3.7	36.9
7	Eggs and omelets	3.6	40.5
8	Other Mexican mixed dishes	3.2	43.8
9	Cheese	3.0	46.7
10	Cold cuts and cured meats	2.2	49.0
	**Hispanic: 25–44 years**		
1	Chicken, whole pieces	9.4	9.4
2	Burritos and tacos	7.2	16.7
3	Beef, excludes ground	5.8	22.4
4	Other Mexican mixed dishes	4.4	26.9
5	Eggs and omelets	4.2	31.1
6	Pizza	3.7	34.8
7	Beans, peas, legumes	2.9	37.7
8	Cheese	2.7	40.4
9	Soups	2.7	43.2
10	Egg/breakfast sandwiches (single code)	2.4	45.6
	**Hispanic: 45–64 years**		
1	Chicken, whole pieces	9.9	9.9
2	Burritos and tacos	6.6	16.5
3	Beef, excludes ground	5.9	22.4
4	Eggs and omelets	4.7	27.1
5	Soups	4.2	31.3
6	Meat mixed dishes	4.0	35.3
7	Fish	3.6	38.9
8	Other Mexican mixed dishes	3.5	42.5
9	Beans, peas, legumes	3.3	45.7
10	Yeast breads	3.1	48.8
	**Hispanic:** **≥65 years**		
1	Chicken, whole pieces	8.6	8.6
2	Soups	5.5	14.0
3	Burritos and tacos	5.0	19.1
4	Eggs and omelets	5.0	24.1
5	Beef, excludes ground	4.1	28.2
6	Fish	3.9	32.1
7	Yeast breads	3.9	36.0
8	Beans, peas, legumes	3.8	39.8
9	Meat mixed dishes	3.4	43.2
10	Pork	2.9	46.2
	**Non-Hispanic White: 18–24 years**		
1	Pizza	8.1	8.1
2	Chicken, whole pieces	7.8	15.9
3	Cheese	4.9	20.7
4	Cold cuts and cured meats	4.2	24.9
5	Burritos and tacos	3.7	28.6
6	Eggs and omelets	3.5	32.1
7	Beef, excludes ground	3.4	35.5
8	Pasta mixed dishes, excludes macaroni and cheese	3.0	38.4
9	Yeast breads	2.7	41.2
10	Burgers (single code)	2.7	43.9
	**Non-Hispanic White: 25–44 years**		
1	Chicken, whole pieces	7.9	7.9
2	Pizza	5.5	13.4
3	Cheese	4.5	18.0
4	Cold cuts and cured meats	4.4	22.4
5	Burritos and tacos	4.0	26.4
6	Beef, excludes ground	3.9	30.3
7	Eggs and omelets	3.4	33.7
8	Meat mixed dishes	3.3	37.0
9	Yeast breads	2.9	39.8
10	Nuts and seeds	2.5	42.3
	**Non-Hispanic White: 45–64 years**		
1	Chicken, whole pieces	6.5	6.5
2	Beef, excludes ground	4.7	11.2
3	Meat mixed dishes	4.2	15.4
4	Pizza	4.1	19.5
5	Cold cuts and cured meats	4.1	23.6
6	Cheese	4.0	27.6
7	Yeast breads	3.8	31.4
8	Eggs and omelets	3.5	34.9
9	Fish	3.2	38.1
10	Nuts and seeds	2.8	40.9
	**Non-Hispanic White:** ≥**65 years**		
1	Chicken, whole pieces	6.3	6.3
2	Yeast breads	5.1	11.3
3	Meat mixed dishes	4.7	16.0
4	Beef, excludes ground	3.9	20.0
5	Cold cuts and cured meats	3.9	23.9
6	Eggs and omelets	3.8	27.6
7	Nuts and seeds	3.3	30.9
8	Fish	3.2	34.1
9	Cheese	3.2	37.3
10	Pork	2.5	39.8
	**Non-Hispanic Black: 18–24 years**		
1	Chicken, whole pieces	11.7	11.7
2	Pizza	6.2	17.8
3	Pasta mixed dishes, excludes macaroni and cheese	4.2	22.0
4	Burgers (single code)	3.9	25.9
5	Chicken patties, nuggets and tenders	3.6	29.5
6	Cheese	3.4	32.9
7	Fish	3.4	36.3
8	Beef, excludes ground	3.1	39.4
9	Eggs and omelets	2.9	42.2
10	Pork	2.8	45.0
	**Non-Hispanic Black: 25–44 years**		
1	Chicken, whole pieces	15.1	15.1
2	Beef, excludes ground	5.1	20.2
3	Pizza	5.0	25.2
4	Fish	4.8	30.0
5	Meat mixed dishes	3.4	33.4
6	Burgers (single code)	3.4	36.8
7	Eggs and omelets	3.3	40.1
8	Cold cuts and cured meats	2.7	42.8
9	Yeast breads	2.7	45.4
10	Pasta mixed dishes, excludes macaroni and cheese	2.6	48.1
	**Non-Hispanic Black: 45–64 years**		
1	Chicken, whole pieces	13.9	13.9
2	Fish	5.8	19.7
3	Yeast breads	4.0	23.7
4	Eggs and omelets	4.0	27.7
5	Burgers (single code)	3.4	31.1
6	Beef, excludes ground	3.3	34.4
7	Pork	3.2	37.6
8	Pizza	3.0	40.6
9	Meat mixed dishes	3.0	43.6
10	Turkey, duck, other poultry	2.4	46.0
	**Non-Hispanic Black:** ≥**65 years**		
1	Chicken, whole pieces	14.6	14.6
2	Fish	5.2	19.8
3	Meat mixed dishes	4.9	24.7
4	Yeast breads	4.8	29.5
5	Beef, excludes ground	4.0	33.5
6	Eggs and omelets	3.9	37.4
7	Pork	3.8	41.2
8	Nuts and seeds	2.3	43.5
9	Turkey, duck, other poultry	2.3	45.8
10	Cold cuts and cured meats	2.2	48.0
	**Non-Hispanic Asian: 18–24 years**		
1	Chicken, whole pieces	15.2	15.2
2	Poultry mixed dishes	4.1	19.3
3	Stir-fry and soy-based sauce mixtures	4.1	23.4
4	Nuts and seeds	4.1	27.5
5	Fish	4.0	31.5
6	Eggs and omelets	3.6	35.1
7	Pizza	3.3	38.4
8	Soups	3.0	41.4
9	Rice	2.9	44.3
10	Beef, excludes ground	2.8	47.1
	**Non-Hispanic Asian: 25–44 years**		
1	Chicken, whole pieces	10.4	10.4
2	Soups	5.9	16.3
3	Fish	4.9	21.3
4	Stir-fry and soy-based sauce mixtures	4.6	25.9
5	Beef, excludes ground	4.6	30.5
6	Rice	4.3	34.8
7	Yeast breads	4.2	39.0
8	Meat mixed dishes	3.4	42.4
9	Eggs and omelets	3.4	45.8
10	Pizza	3.3	49.0
	**Non-Hispanic Asian: 45–64 years**		
1	Soups	8.8	8.8
2	Fish	8.6	17.4
3	Chicken, whole pieces	6.4	23.7
4	Rice	5.2	28.9
5	Yeast breads	4.4	33.3
6	Stir-fry and soy-based sauce mixtures	4.0	37.3
7	Pork	3.7	41.0
8	Fried rice and lo/chow mein	3.2	44.1
9	Meat mixed dishes	3.1	47.2
10	Nuts and seeds	3.0	50.2
	**Non-Hispanic Asian:** ≥**65 years**		
1	Soups	8.4	8.4
2	Fish	7.6	16.0
3	Chicken, whole pieces	6.4	22.4
4	Rice	5.0	27.4
5	Stir-fry and soy-based sauce mixtures	4.9	32.2
6	Yeast breads	4.8	37.0
7	Pork	4.2	41.2
8	Nuts and seeds	3.8	45.0
9	Meat mixed dishes	3.5	48.5
10	Eggs and omelets	3.4	51.9
	**Other: 18–24 years**		
1	Chicken, whole pieces	9.1	9.1
2	Beef, excludes ground	8.0	17.0
3	Burritos and tacos	5.4	22.4
4	Chicken patties, nuggets and tenders	4.9	27.3
5	Pizza	4.9	32.2
6	Milk, reduced fat	3.4	35.6
7	Cheese	3.3	38.9
8	Pasta mixed dishes, excludes macaroni and cheese	3.3	42.2
9	Eggs and omelets	3.2	45.4
10	Other Mexican mixed dishes	2.9	48.3
	**Other: 25–44 years**		
1	Chicken, whole pieces	10.4	10.4
2	Beef, excludes ground	7.2	17.6
3	Pizza	6.6	24.2
4	Cold cuts and cured meats	3.5	27.7
5	Burritos and tacos	3.4	31.0
6	Eggs and omelets	3.3	34.3
7	Fish	3.1	37.4
8	Nuts and seeds	2.8	40.2
9	Meat mixed dishes	2.7	42.9
10	Cheese	2.6	45.5
	**Other: 45–64 years**		
1	Chicken, whole pieces	10.2	10.2
2	Yeast breads	4.2	14.4
3	Nuts and seeds	4.0	18.4
4	Meat mixed dishes	4.0	22.3
5	Fish	3.9	26.2
6	Pasta mixed dishes, excludes macaroni and cheese	3.8	30.1
7	Burgers (single code)	3.7	33.7
8	Beef, excludes ground	3.4	37.1
9	Chicken patties, nuggets and tenders	3.4	40.5
10	Turkey, duck, other poultry	3.2	43.8
	**Other:** ≥**65 years**		
1	Fish	6.2	6.2
2	Other Mexican mixed dishes	5.6	11.8
3	Yeast breads	5.4	17.3
4	Eggs and omelets	4.8	22.1
5	Chicken, whole pieces	4.0	26.0
6	Cheese	3.7	29.7
7	Cold cuts and cured meats	3.6	33.3
8	Milk, reduced fat	3.6	36.9
9	Meat mixed dishes	3.5	40.4
10	Beans, peas, legumes	3.3	43.7

## Discussion

This study identified the top ten dietary sources of protein by age and race/ethnicity groups. The top ten sources of protein accounted for over 40% of dietary protein irrespective of race/ethnicity or age category, having major implications for the sustainability of our nation's food supply (e.g., greenhouse gas

emissions, nitrogen and phosphorous pollution, biodiversity loss, and water and land use) (19). This analysis is aligned with local and national activity around nutrition improvements to reduce chronic disease risk. Findings from this analysis contribute to guidance from expert committees suggesting interventions might target (1) increasing protein intake, exclusively among older adults (≥65 years); (20) (2) substituting nutrient-rich sources of protein intake (i.e., beans, peas, legumes) for sources demonstrating deleterious health effects (i.e., cold cuts and cured meats); (21) and (3) improving the sustainability of the nation's food supply (19).

While prioritization has been placed on increasing protein intake among older adults (22, 23), limited evidence to monitor intake suggests no change, which may in part be due to policies that by design may not reach all sub-populations. From 2001 to 2014, protein intake ranged from 14 to 16% of calories irrespective of age and gender category (5). On average, 30% of protein consumed in the United States is derived from plant sources (24). Determining which foods contribute to dietary protein intake within each racial/ethnic and age group could allow for the development of culturally adapted public health messages. In addition, determining specific food contributions within each racial/ethnic and age group category allows health practitioners and dietitians to make informed and valuable recommendations on healthy protein sources. In order to better understand the disparities in obesity and risk factors associated with chronic disease among racial/ethnic groups we must examine the dietary contributions, specifically from dietary protein.

Traditionally it has been thought that animal protein contributes significantly to the development of chronic disease (e.g., cardiovascular disease [CVD], type 2 diabetes [T2D], hypertension), whereas plant-based protein may be more protective. Though the evidence for animal and plant-based protein contributing to chronic disease are mixed (7, 25–28), replacing energy-rich, nutrient poor sources of animal protein with a different type of animal protein, or with beans, peas, and legumes, would have positive effects on the population's health (19, 29).

Furthermore, having only two racial/ethnic groups with plant-based sources of protein in their top ten has major implications on the sustainability of the nation's food supply in addition to the nation's health (19, 30). Animal-based protein is associated with greater greenhouse gas emissions when compared to plant-based protein (31, 32). The top ten sources of protein contributed to over 40% of dietary protein consumption. Increased consumption of meat puts increased pressure on farmers to produce enough supply to meet the demand, which in turn strains resources, including water, land, and feed. Additionally, when local farmers cannot meet the supply, meats are imported from distant locations within and sometimes outside of the United States, which increases energy consumption due to the fuel required to refrigerate and transport the products (19). In conclusion, while a key recommendation of the 2015 Dietary Guidelines is to consume “a variety of protein foods in nutrient-dense forms,” (21) these data suggest several of the subgroups named (i.e., legumes) are underconsumed. Protein foods are important sources of other nutrients, including B vitamins, selenium, choline, and zinc, but nutrients provided by various types of protein foods differ (33–35). For this reason, the latest Dietary Guidelines provide subgroup recommendations for the following protein sources: seafood; meats, poultry, and eggs; and nuts, seeds, and soy products.

The strengths of this study include a sample representative of the US non-institutionalized population and an oversample of Asian Americans allowing for comparisons of sources of protein intake among Asian Americans to other race/ethnic groups. Stratifying by race/ethnicity helps us to better understand factors accounting for differences in protein intake in the US population. There are several limitations that should be noted. A single dietary recall cannot represent an individual's usual intake and has measurement error; however, the NHANES multiple-pass method demonstrated acceptable validity and reliability when compared with urinary nitrogen, a recovery biomarker of protein intake (36, 37). Although rigorous methods were used for dietary recall that allows for the incorporation of diverse dietary patterns, data are subject to measurement error (i.e., recall and coding errors). Furthermore, the race/ethnicity categories are broad and one race/ethnic category was not directly sampled (e.g., Other [includes mixed race]). As such, this precludes the ability to distinguish between subpopulations (i.e., Chinese, Indian, Filipino), potentially masking disparities by ethnicity and country of origin (38, 39).

This analysis highlights racial/ethnic and age differences in top sources of protein, which is important for developing targeted efforts to increase protein intake among high-risk subpopulations. Public health strategies that encourage diversity in protein sources in food preparation and incorporate legumes and nuts along with poultry have the potential to shift the overall population protein intake distribution toward improving overall diet quality.

## Data Availability Statement

The raw data supporting the conclusions of this manuscript will be made available by the authors, without undue reservation, to any qualified researcher.

## Ethics Statement

The studies involving human participants were reviewed and approved by The Research Ethics Review Board at the National Center for Health Statistics. The patients/participants provided their written informed consent to participate in this study.

## Author Contributions

JB conceived of the study. MF conducted the data analysis, CP and JB drafted the manuscript, and all authors edited the manuscript. All authors read and approved the final manuscript.

## Conflict of Interest

The authors declare that the research was conducted in the absence of any commercial or financial relationships that could be construed as a potential conflict of interest.

## Abbreviations

CVD: cardiovascular disease
T2D: type 2 diabetes
FNDDS: Food and Nutrition Database for Dietary Studies
NHANES: National Health and Nutrition Examination Survey
NHA: non-Hispanic Asian
NHB: non-Hispanic Black
NHW: non-Hispanic White
RDA: Recommended Dietary Allowance
USDA: United States Department of Agriculture.
